# Empirical Study of a Room-Level Localization System Based on Bluetooth Low Energy Beacons

**DOI:** 10.3390/s21113665

**Published:** 2021-05-25

**Authors:** Pedro J. García-Paterna, Alejandro S. Martínez-Sala, Juan Carlos Sánchez-Aarnoutse

**Affiliations:** Department of Information and Communication Technologies, Universidad Politécnica de Cartagena, 30202 Cartagena, Spain; pedro.gpaterna@edu.upct.es (P.J.G.-P.); juanc.sanchez@upct.es (J.C.S.-A.)

**Keywords:** indoor positioning, room-level, Bluetooth Low Energy, BLE beacons, fingerprinting

## Abstract

The ability to locate an object or a person at room-level inside a building or a house could have multiple applications. In this study, we adapt the fingerprint technique using Bluetooth Low Energy to locate the exact room of a person, seeking a simple and low-cost solution. The system is based on BLE beacons deployed at fixed positions and a person carrying a BLE scanner that generates fingerprints from the BLE beacons in coverage. We formulate it as a classification problem where each room is a class; the objective is to estimate the exact room, trying to maximize the area and number of rooms, but also trying to minimize the number of BLE beacons. The room estimation engine is based on a kNN (k-nearest neighbors) classifier. We evaluate the accuracy in two real scenarios and empirically measure the room estimation success related to the number of BLE beacons. As a proof-of-concept, a laptop and a Raspberry Pi are used as BLE scanners to test different hardware. We follow a measurement campaign for several days at different times to evaluate the stability and repeatability of the system. With just a few beacons an accuracy between 70 and 90% is achieved for house and university scenarios.

## 1. Introduction

There are indoor location value-added services that do not require an (x,y) coordinates resolution [1,2]. The ability to locate an object or a person at room-level inside a building could have multiple applications and can drive the development of innovative smart home or smart building services [3]. For instance, an indoor occupancy monitoring system could be used to detect human presence, and to count people in target areas. In this regard, Rueda et al. [4], and Sun et al. [5] presented comprehensive reviews of building occupancy estimation and detection systems, mainly based on sensor fusion. Moreover, in the occupancy problem, many works have been proposed to address specific use cases: safety and security [6], space allocation in commercial building [7], and energy management [8]. In addition, there is presently an important concern for occupancy monitoring in public buildings due to SARS-Cov-2 control. These use cases are examples where a room-level location system based on wireless technology (Bluetooth Low Energy, Wifi, UWB, or Zigbee) can be applied [1,9]. 

Due to the widespread of Bluetooth Low Energy (BLE) technology [10], its low energy consumption, and low cost, we propose a BLE system that provides a room-level location service. We also describe the design, installation, and testing methodology of the system, which has been developed looking for simplicity, ease of installation, development, maintenance, and low cost. In our work, we assume that the person to be localized wears a BLE-enabled smart device. As a proof of concept, we use a laptop and Raspberry Pi 4, but our proposal can be extrapolated to other smart devices such as IoT wearables or smartphones.

Bluetooth Low Energy standard defines two transmission types: data and advertising. Advertising transmission uses three physical RF channels for discovering devices, initiating a connection, and broadcasting data. Data transmission uses specific RF channels for communication between connected devices. As defined in the standard, an advertising message has a header and a payload between 0 and 31 bytes. The iBeacon message is a proposal from Apple that adopts the BLE standard but specifies its own message format embedded in the payload [11]. Nowadays, the following BLE standards are available; 4.0, 4.1, 4.2, 5.0, and the recent 5.1 [12,13]. The basic advertising mechanism, message format, and the scanning process are the same for all the standards, allowing compatibility between available commercial products. Bluetooth 5.1 defines a new mechanism for the angle of arrival measurement that will improve indoor location systems, but it is needed that the beacons and the scanner follow the direction-finding property. Therefore Bluetooth 5.1 angle-of-arrival is not backward compatible with the previous standards and available BLE devices and it is not considered for our system.

The basic elements of a BLE system are BLE beacons and BLE scanners [10]; a BLE beacon periodically broadcasts advertisement messages at a configured time interval and transmission power. A BLE scanner turns on its radio in receiving mode, monitors the advertising channels, and receives advertisement messages from the surrounding BLE beacons in coverage. Moreover, a BLE scanner is able to measure the received signal level from each beacon or RSSI (Received Signal Strength Indicator) commonly expressed in dBm. Current BLE localization services [10,11] are mainly based on the acquisition of the RSSI data from the advertising messages transmitted by a beacon. 

Three different techniques are used for such RSSI-based location: lateration, angulation, and fingerprinting [3]. The fingerprint approach is widespread from Wi-Fi [14] to BLE systems [15] for two main reasons: (1) its simplicity; (2) it is possible to achieve a bounded positioning error of few meters. The main drawback of these systems is that they require a lot of manpower and time to build the reference radio map on a point grid [16,17]. During the calibration step, RSSI measured from beacons in coverage are collected at each reference point to generate the so-called calibration fingerprint vector. Each calibration fingerprint vector is labeled with the ID point and its Cartesian coordinates from a 2D map to build the calibration fingerprint database. Then, when a fingerprint is collected from a device and sent online, it is processed by a positioning algorithm that compares the signal pattern of the intended fingerprint with the calibration fingerprint database to infer the (x,y) position [1,14]. 

The proposed system is based on the well-known fingerprint technique using commercial BLE beacons deployed at fixed positions in the scenario, and a person carrying a BLE scanner (which is the target to be localized) but, instead of localizing the target at grid point inside a room on a 2D plane [18,19], our system estimates the room where the person is. The BLE scanner periodically scans for advertisements of the surroundings BLE beacons and generates a fingerprint from a time interval. The fingerprint is formed by the average RSSI from the advertisements received from each BLE beacon during that time interval. Then, the BLE scanner sends the fingerprint to a server for storing and processing the data. 

As our goal is to locate at room-level, we formulate the localization problem as a classification problem where each room is a class characterized by the calibration fingerprints from the room. In [20] Lovón-Melgarejo et al. present a review of machine learning algorithms applied to a BLE location system. One of the conclusions of this work is the good trade-off among accuracy, simplicity, and ease of use of the k-nearest neighbor (kNN) algorithm. Therefore, as a proof-of-concept, our room-level localization system is built upon a kNN classifier whose input is the instant fingerprint from a BLE scanner, and the output is the estimated room in which the related person is located. As stated before, a laptop and a Raspberry Pi are employed as BLE scanners carried by a person in order to evaluate different hardware. Notice that the signal pattern and signal strength measured by the BLE radio from different devices may differ due to radio hardware heterogeneity and differences in the radiation patterns and the respective antenna gains [21]. This is a generalized and challenging problem in all RSSI-based indoor positioning systems [1,14]. 

Continuing with the related work, most of the previous indoor positioning systems are intended to grid points at a 2D plane [1,2] but are the foundation of room-level positioning systems. A different approach is based on proximity systems [11], where the goal is to detect how far a target is from a point of interest; in [21] a BLE proximity system is analyzed. In that work, the factors that affect the signal quality and produce the distortion of the signal pattern of a BLE fingerprint are also studied (user orientation, mobility, obstacles, RF channel multipath, and fading effects, to name a few of them, are relevant for any indoor positioning system).

As discussed in [14], there are indoor positioning systems that propose the fusion and processing of wireless signals with the data from sensors from a smart device (such as the accelerometer data), or people movement filters based on the map topology knowledge; but we propose to study the feasibility of using only BLE technology for a room-level positioning system with the minimum hardware needed. Furthermore, our proposal may be improved and optimized by adding these extra layers from more advance indoor positioning systems.

In [22] a motion filter based on a transition probability between zones is applied. The proposals of [23,24] are also room-based, but they place BLE beacons on the ceiling of each room and the test scenarios are small (with only three rooms in both cases). In our work, we install the beacons on the wall and we try to minimize the total number of beacons in the test scenarios (which are significantly bigger).

In [25] the authors use beacons with different transmission power but attached to a person and the BLE receivers act as scanners and are installed at fixed positions. Hence, this is a different architecture of our system. In [26] the authors use custom BLE beacons and focus on a home energy management system. Finally, in [27], the authors proposed an interesting system for occupancy monitoring of smartphones using BLE for indoor and outdoor areas, but the main concern is on open data availability. 

To summarize, our main contributions are:

- a simple and low-cost architecture for a room-level indoor positioning system based on Bluetooth Low Energy is proposed;
- the design decisions are provided and explained in detail, remarking key ideas and lessons learned to reproduce the solution;
- a simplified procedure and efficient time-consuming process to calibrate and test a scenario is proposed;
- the system is tested and evaluated in two real scenarios. The relation of the number of BLE beacons and the room estimation success is empirically analyzed.

The rest of this paper is organized as follows. The system details are introduced in Section 2. Section 3 discusses the experiments of two testbeds and describes and provides the corresponding results. In Section 4 and Section 5, discussions and conclusions with final considerations and remarks are drawn.

## 2. System Description

**Figure 1:**
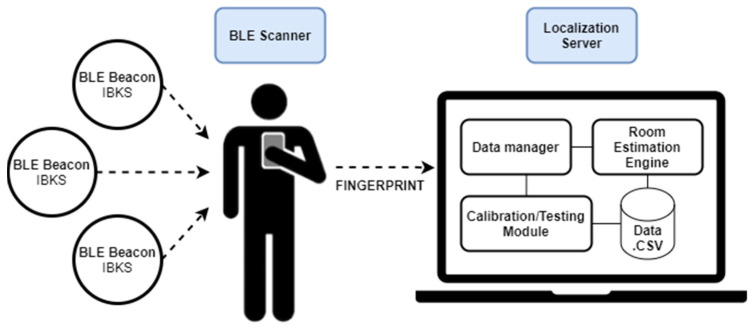
Overall system.

**Figure 2:**
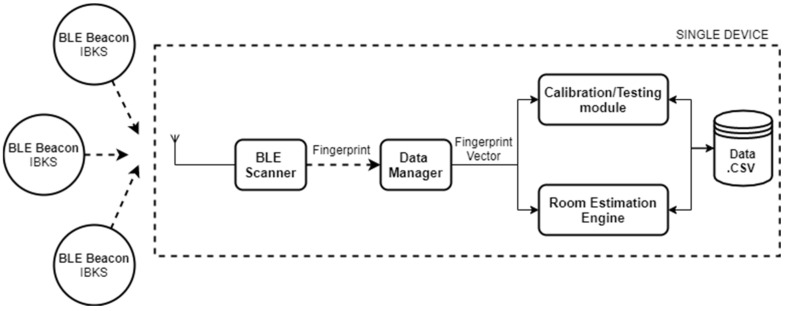
System architecture on a single device.

The proposed room-level localization system is based on the fingerprinting technique, in which the BLE beacons are placed at fixed positions in the use case scenario, while a person carries a mobile device with the role of a BLE scanner. The overview of our system is shown in Figure 1 which represents the elements of a generic system; the BLE scanner attached to a person which periodically scans for location BLE beacons, builds an instant fingerprint vector, and sends it to the location server. On the other side, Figure 2 shows the system architecture running on a single device, i.e., in our work the BLE scanner and location server are running on a laptop with Linux for the sake of simplicity, as it will be explained in detail in the next points.

The room estimation engine was implemented with Matlab scripts and it ran the kNN classifier and calculated the performance metrics of the system. The rest of the software modules were implemented in Python 3 and were executed on Linux operating system. In our Linux implementation for the BLE scanner module, the Bluez library [28] was used to manage the Bluetooth radio of the person’s device.

Before explaining, in detail, the overall system and its components, it is convenient to distinguish the steps required for the implementation of the system in a real scenario, as well as the operating modes of the system itself. The deployment cycle of this system was adapted from previous work [17], consisting of the following six steps:

1. Pre-project and planning phase; in which the map layout, the zoning, and their sizes are studied. ID zones are assigned. The number of BLE beacons and their position on the map is planned. Each beacon on the map is assigned with a simple and unique ID from 1 to 254 in decimal. The result is a deliverable with the zoning map where the planned beacons and their unique IDs are represented.
2. Configuration and deployment of BLE beacons; each BLE beacon is configured according to the previous planning phase. Next, according to the map, the beacons are fixed on walls by means of stickers. It is suggested to place them at a height between 2 and 2.5 m, as high as a person can get without using a ladder. Notice that beacons are approximately positioned, there is no need to use tools or exact coordinates. Therefore, this step can be done quickly.
3. Fingerprint calibration phase; the signal pattern of the beacons has to be characterized for each room in order to build the fingerprint database. A BLE scanner device is used as a reference, in our case a Dell laptop running Linux. The calibration process used to be very time-consuming in other proposals [16]. We suggest a quick calibration procedure for a few minutes per zone in which the user collects fingerprints with raw data labeled with the actual ID zone. We recommend a normal movement of the user around the room, sometimes stopping in a position for a few seconds and changing direction or orientation. Notice that it is important to reproduce the usual mobility pattern and behavior of a person during a few minutes; as a room is considered a class in our problem, the labeled fingerprints characterized the room. This is not a time-intensive process and from our experience, a mean time of around 2 min is enough to calibrate a room.
4. Testing phase; this stage is intended to obtain labeled data during several days with different users and hardware mobile devices acting as BLE scanners. Again, attention should be paid to reproduce the usual mobility pattern of a person around the room. These simple data provide a ground-truth and should be enough to test performance and to tune the room estimator.
5. Online phase; once the system performance has been measured and the room estimator has been tuned and validated, the system may go into production. Therefore, online fingerprints are processed by the room estimator engine in real-time.
6. Maintenance; a BLE beacon may fall off the wall or run out of battery, therefore, it is convenient to periodically check the system. The location server may implement a daily checking process and trigger an alert if no data has been received from a BLE beacon during a time period.

Concerning the operating modes of the system, the following paragraphs overview the three main modes:

- Calibration mode; used to characterize the signal pattern from each room or zone and to generate the corresponding calibration fingerprint. In this mode, the room estimator engine is not invoked, only labeled data are collected for calibration.
- Testing mode; this mode is really close to calibration mode, but it is intended to collect actual fingerprints labeled with the true ID zone (introduced by the user). This way, a measurement campaign with different BLE scanner hardware, users, or time, can be designed to systematically collect data. Afterward, these data will be used to rigorously evaluate the performance under controlled conditions. In this mode, the room estimation engine is invoked offline yielding performance metrics.
- Online mode; once the system is calibrated, tuned, and validated, the online mode periodically sends the captured fingerprints to a location server. The location server invokes the room estimator engine online, which may have an integration interface with an application server, such as energy management, occupancy monitoring, etc.

Since our main objective is to demonstrate the feasibility of a room-level localization system using the minimum number of BLE beacons, from this point, the rest of the paper is only focused on calibration and testing modes, as well as for performance evaluation.

The proposed system should facilitate the implementation of the BLE scanner in any kind of device. To this end, first of all, an appropriate BLE beacon configuration should be chosen. After considering the different available options, the iBeacon format was selected due to its widespread commercial adoption and its easy configuration [11]. Besides, nowadays there are a vast number of API libraries in the most popular programming languages and platforms (from embedded computers to IoT devices or smartphones) can be used to easily scan and process iBeacon messages. 

An iBeacon advertising packet provides several fields that are useful to identify and classify each beacon. The main fields of an iBeacon message are UUID, Major and Minor, which the user may configure by means of an App. In our case, all the beacons involved in the location service are configured with a particular value at the 16-byte UUID field, called UUID *LOC* value. As there may be a crowded environment of BLE devices broadcasting their advertisement packets, this fixed UUID value is used to filter the received advertisements at the BLE scanner, recording, and processing only the raw data from the intended location BLE beacons. In addition, the Major field from the iBeacon message (two bytes) is used to encode a simple ID for each BLE beacon of a scenario. Notice that a BLE device has its own MAC address, which is usually set by the manufacturer. The MAC from a BLE beacon could be used as a valid beacon ID, but it would complicate the deployment and maintenance process. With our proposal, it is not necessary to know or to register each MAC from a BLE beacon at the location server, it is only necessary to configure each BLE beacon with the ID planned in the map. As we will explain later, the fingerprints are efficiently built using these IDs. In case a BLE beacon needs to be changed, we just need to configure the iBeacon message with the *UUID LOC* value and the Major field with the ID according to the map defined during the pre-project and planning phase. We consider it is a simple and time-saving procedure. 

At the hardware level, the iBKS105 commercial beacons [29] from Accent Systems were chosen. These beacons are based on a Nordic Semiconductor nRF52832 chipset and use a high-capacity button cell battery for long life durability. The company provides a configuration App, which allows quick detection of an iBKS105 beacon and an easy configuration of the main parameters. In our use case: transmission interval of advertisements, the transmission power, and the main iBeacon fields; UUID with the *UUID LOC* value, and Major as the beacon ID.

On the other hand, the person must carry a mobile device acting as a BLE scanner. This can be a tablet, smartwatch, smartphone, wearable, or other BLE-enabled devices. In this work, as a proof-of-concept and, pursuing to simplify the setup and ease the calibration and testing processes, a Dell laptop and a Raspberry 4 were used to check the hardware heterogeneity effect. The simple reason is that both devices can run the main software modules at the same time; therefore, they can simultaneously behave like the person’s BLE scanner and the server that collects and stores the fingerprints. This way, to emulate a new scenario is reasonably fast and does not depend on having a Wi-Fi access or connection to the server (since it is integrated into the same equipment). 

### 2.1. BLE Scanner and Fingerprint Generation 

**Figure 3:**
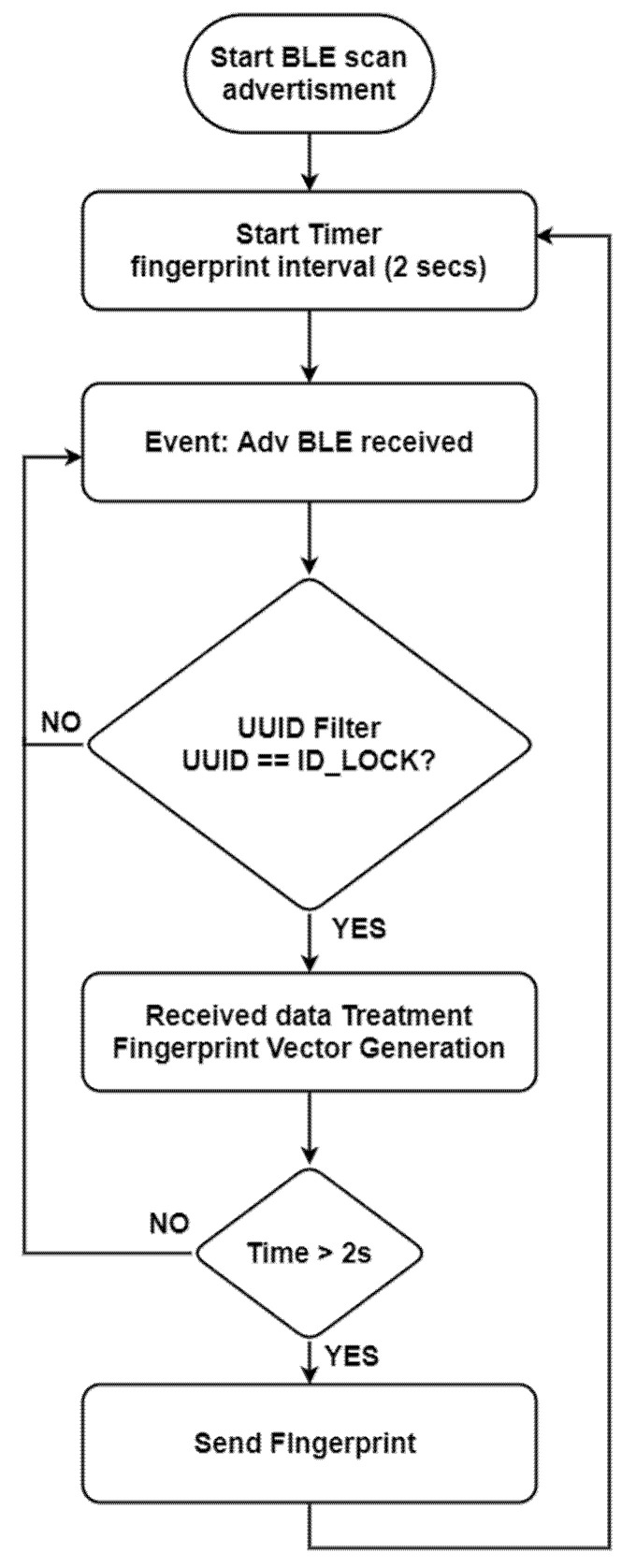
BLE scanner module flowchart.

As introduced before and depicted in Figure 1 and Figure 2, a person must carry a device with the role of a BLE scanner intended for the fingerprint generation. Figure 3 shows the flowchart of the BLE scanner which is responsible for (1) receiving all BLE advertising of the surrounding beacons and filtering the ones from the location BLE beacons, (2) summarizing these raw data into small amounts of meaningful information called a fingerprint, during a time window of two seconds, and (3) sending this fingerprint via MQTT protocol to the data manager module at the location server. 

The BLE scanner builds the fingerprint during the time window with just the location beacons in coverage, i.e., only the detected beacons and filtered by the *UUID LOC* value, forming an array of variable sizes. For each location beacon detected, there are three fields in the array:

- ID beacon i. From the iBeacon’s Mayor field, the ID of the beacon i is obtained.
- ADV counter i. Counter of advertising frames received from beacon i.
- RSSI i. Average RSSI value from the received advertising frames from beacon i.

Therefore, a fingerprint sent by the BLE scanner to the location server is an array of variable sizes with just the data recorded from the location beacons during the time window interval. Notice the BLE MAC is not used, and it is a simple procedure that saves memory and it is easy to implement in IoT or resource-constrained devices, such a BLE wristbands. When the device sends the fingerprint, it adds an ID device field related to a person. 

### 2.2. Data Manager Module and Fingerprint Vector 

The Data Manager runs on the location server-side and it receives all the fingerprints sent by the BLE scanners stored in a queue. The Data Manager must be configured with the beacon list which contains the IDs of the available beacons according to the map. This beacon list should be defined just once time, after the planning stage. From each fingerprint waiting on the queue, the Data Manager processes them to build the corresponding final fingerprint vector. 

A high signal level received from a BLE beacon would be around −30 to −50 dBm [21]. The lower signal level that a BLE radio is able to distinguish from noise floor and capable of reconstructing a message without errors is called the radio sensitivity. The value of that radio sensitivity is around −100 dBm. At the sensitivity threshold, the packet reception rate of advertisements is barely low but may happen. Under the sensitivity threshold, the radio is not able to receive any transmission. 

The final fingerprint vector is the input for the room-level classifier and reflects the signal pattern feature space. Due to that reason, when there is missing data from a beacon at the fingerprint, it is needed to complete this data at the final fingerprint vector and it is decided to use the sensitivity threshold as an approximation.

To clarify, it has to be distinguished between the fingerprint sent by a BLE scanner, which may have not sorted data or may have a lack of RSSI data due to undetected beacons, and the final fingerprint vector, which has data from all the beacons sorted by their IDs, even those undetected beacons filled with the sensitivity threshold of −100 dBm. 

Remember that the fingerprint vector is related to a time window of two seconds and must correspond with the positioning of a person in a specific room. The next fingerprint vector from a device may correspond to the same room or to another room.

### 2.3. Calibration and Testing

The fingerprint technique requires to build a reference radio-map, so-called calibration fingerprint, where the labeled fingerprint vectors with the actual position are recorded [14,15]. Since we formulate the room estimation process as a classification problem, it is needed to characterize a room as a class with its corresponding fingerprint vectors. 

The calibration module allows to perform a calibration of a specific room and to label all the collected fingerprint vectors with the ID room. When the Data Manager module is running, it is continuously sending fingerprint vectors when available. A simple user interface is required where the user can introduce a user ID. Additionally, we found it useful to set the time for how long the calibration recording lasts per zone and a start command; the explanation is to get a balanced dataset per all the classes. As a result, the same number of fingerprint vectors for each room are recorded equalizing the dataset. Therefore, during a room calibration period, the calibration module is receiving fingerprint vectors from the Data Manager module and it stores these data in text file CSV alike adding the fields of ID user, and ID actual zone. The text file follows a table format where a row is related to a fingerprint vector; assuming that N is the number of deployed location beacons, the first N columns correspond to the average RSSI from each beacon in a fingerprint. Then, the N + 1 column is the device ID, and the last column is the actual room ID. The calibration process ends when all the zones have been traversed. Finally, a CSV file, called calibration fingerprint, with the data obtained is generated and stored in memory. Table 1 shows an example and extract of a CSV file from the calibration fingerprint with the laptop (Dev. ID 1) from the house scenario; each fingerprint vector has six elements related to the RSSI from six beacons (B1 to B6). The table contains four fingerprint vectors from two rooms: rows #1 and #2 from Room ID 4, and rows #3 and #4 from Room ID 3.

Regarding the testing module, it has to be remarked that it is implemented by the calibration module, however, with a different role. Conceptually, we explain as distinct modules to emphasize the nonidentical purposes, but it follows an equal working principle. Due to that, the testing module is used to gather ground-truth fingerprint vectors labeled with the actual ID zone. The testing module used to implement a systematic measurement campaign under controlled conditions, as is explained in detail in the next section, is useful to perform a system evaluation and analyze the success room estimation rate. Notice that the calibration and testing files are CSV alike and follow the same format for a target scenario. 

To conclude, there is a single module with two roles, calibration or testing, which is a tool to collect labeled data, but it is compelling to reflect on the procedure to perform. As stated previously, each room is a class characterized by its particular fingerprint vectors. Therefore, it is crucial to reproduce the usual mobility pattern and behavior of a person during a few minutes; moving around the room, stopping for a few seconds and walking again, and changing the user’s orientation. We come to the conclusion that our proposal is an efficient and not time-intensive process, easy to reproduce that allows gathering real and generalizable signal patterns from the surrounding beacons.

### 2.4. Room Estimation Engine

The room estimation engine is composed of the room estimation module and the performance metrics module. The input of the room estimation module is a fingerprint vector from a device and the output is the estimated room for a user. As a fingerprint vector corresponds to a measurement time interval of two seconds, we make the assumption that during this lapse of time the user belongs to a class, i.e., the user stays in a particular room. Therefore, for each fingerprint vector, the room estimation module is invoked, and the corresponding estimated room is obtained. 

Hence, a classification algorithm is needed to resolve our problem. As stated in the introduction section, there is a review of machine learning algorithms applied to a BLE location system in [20], where the authors found a good trade-off among accuracy, simplicity, and ease of use of the kNN algorithm. Consequently, as a proof-of-concept, the kNN algorithm is used as the classifier algorithm for our system. The working principle of the kNN classifier adapted to the room estimation problem is described in detail next:

- there is a calibration fingerprint file made by a reference device. Each room is a class composed of fingerprint vectors labeled with the actual ID room;
- for an instant fingerprint vector from a device to be classified, the similarity between the instant fingerprint vector and each calibration fingerprint is calculated. The similarity is measured by means of the Euclidean distance metric;
- a list with the Euclidean distance sorted by the weight of that metric is obtained. It is assumed that the smaller the weight, the higher the similarity between a specific calibration fingerprint and the instant fingerprint vector;
- then, the k nearest calibration fingerprints are selected to vote the class. In this context, nearest has the meaning of similarity between signal vectors. In another way round, the k most similar calibration fingerprints (minor weights) are selected according to the instant fingerprint vector;
- finally, as the selected calibration fingerprints are labeled with their corresponding room, there is a majority voting process to elect the room. An odd number of k was chosen in order to avoid tie to voting situation. To clarify, as the k elected calibration fingerprints have the same voting weight, the most repeated room will be the one estimated by the classifier.

In our case, by tuning the kNN classifier it is found that k equals 5 is optimal to achieve a high success room estimation rate, as it is explained in the next section. 

In addition, this module allows two execution modes: online and offline. The online mode would be the usual mode of operation of the system. In this mode, the room estimation module is invoked every time a fingerprint vector from a device is received, then estimating a room in real-time.

Regarding the offline mode, its main purpose is to focus on the performance evaluation, easing the tuning of the kNN classifier, and analyzing as fast and simple as possible a session test from a user. The offline mode does not have any data reception routine, instead, the module loads the necessary CSV files; calibration fingerprint file, and session test file. Concerning a session test file, as explained before, it has the same format as the calibration fingerprint with fingerprint vectors labeled with ground truth, i.e., the actual ID room. Due to this, each time the kNN room classifier (or room estimation module) is invoked, a results file is generated, where a column is the actual ID room and the next column is the predicted ID room. Each row of the results file corresponds with a fingerprint vector from the session test file. When all the fingerprints from the test session have been classified, then the results file is ended. Next, the results file is processed by the performance metrics module from the room estimation engine.

One of our research goals is to analyze the relationship between the room success estimation and the number of BLE beacons. As the beacons are deployed in a use case scenario and form the features of the signal pattern, we wonder if a signal pattern composed of just a selection of beacons would work out properly. To this end, a parameter at the kNN classifier is defined, called beacon mask, that acts as a mask to select which beacons are used on the signal pattern. This issue will be explained in detail with examples in the next section.

#### Performance Metrics for System Evaluation

The results file from a session test is processed by the performance metrics module to evaluate the system. Since our room-level positioning system can be formulated as a multiclass classification problem where each room is a class, the goal is to maximize the classification success, therefore the following concepts and multiclass metrics can be used to evaluate the performance of the system [30,31]; confusion matrix, precision, recall, f1-score, accuracy, macro-precision, macro-recall, and macro-f1 score.

The first measure to be defined is the confusion matrix since it allows to define of other performance-related metrics. In the case of room localization, the classifier must assign a room to each test fingerprint. The prediction can be correct (it is assigned to the correct room), or incorrect (the fingerprint is assigned to another room). A confusion matrix accumulates all the predictions, crossing (for all classes) the predicted class with the actual class for all the classes (rooms). Each column represents the number of predictions of each class, while each row represents the instances of each true class. As can be intuited, an ideal system returns a diagonal matrix (the predictions coincide with the true class, while the rest of the elements have zero value). Obviously, real systems are not perfect, so this matrix allows us to visualize which classes (rooms) are misinterpreted by the classifier, i.e., room estimation module.

Precision (for room *i*) represents the number of True Positives (the number of fingerprints correctly classified to the room *i*) out of all the predictions for the room *i* (True and False positives).

(1) $$Precision_{i}=\frac{True\;Positives_{i}}{True\;Positives_{i}+False\;Positives_{i}}$$

Recall (for room *i*) indicates the number of True Positives for room *i* out of all the actual fingerprints that belong to the room *i* (that is, the number of correct predictions for that room plus all the times that predictor has not assigned the room, but it was true). A higher number of false positives leads to lower values. This occurs when the classifier makes erroneous predictions, assigning to another room a value that really belongs to the room under study.

(2) $$Recall_{i}=\frac{True\;Positives_{i}}{True\;Positive_{i}+False\;Negatives_{i}}$$

Another more complex metric is the F1-score for room *i*, which is defined as the harmonic mean of precision and recall for the room *i*. Theoretically this metric reaches its maximum at 1 (infallible model), and its minimum at 0 (the classifier fails all the time). The value of this metric should be analyzed carefully and considering precision and recall trends.

(3) $$F1-score_{i}=2\times \frac{Recall_{i}\times Precision_{i}}{Recall_{i}+Precision_{i}}$$

With regard to global metrics of the overall system (without particularizing into classes), we will first focus on accuracy. For a testing session, with a balanced dataset of fingerprints from all the rooms, that metric is the rate between the correctly estimated fingerprints out of all the testing fingerprints:

(4) $$Accuracy=\frac{TP+TN}{TP+TN+FP+FN}=\frac{Number\;of\;success\;fingerprints}{Total\;test\;fingerprint}$$

Finally, the last global metrics to assess the whole room-level localization system are:

- macro-precision is the arithmetic mean of the per-room precision;
- macro-recall is the arithmetic mean of the per-room recall;
- macro-F1 is the arithmetic mean of the per-room F1-scores.

## 3. Testbeds and Results

The room-level localization system and methodology described in the previous section were tested in two real scenarios: a terraced house and a section of the basement of one of our university buildings.

Firstly, it is necessary to estimate the number of beacons and to plan their location on the map. For this planning phase, the number of zones and their dimensions were analyzed on the floor plan aiming to choose where to deploy approximately each BLE beacon in a wall. As a proof-of-concept, we approximated the maximum coverage distance of a beacon to a few meters taking into account (1) the transmission power of a beacon, (2) an average signal loss of 3 dB per wall, and (3) the user’s worst-case orientation (average loss of 3 dB) [3,21]. Then, based on these considerations, we heuristically set the BLE beacons’ locations on the map, trying to establish intersection zones formed by at least three BLE beacons. In addition, unlike other works in which the beacons are placed on the ceiling [19,24], we consider placing the beacons only on the wall (which implies a restriction in the deployment).

The purpose of this methodology is twofold: on the one hand, it facilitates the quick installation of the BLE beacons, and on the other hand, the beacons are easily accessible, thus their batteries can be replaced without a ladder. All BLE beacons were placed on walls as high as possible (between 2 and 2.5 m). Notice that this methodology does not require defining the exact position or coordinates of a BLE beacon in a map (and consequently in a real scenario), neither it is not needed any meter nor tool. We consider that is a generalizable and simple procedure for a quick deployment regardless of the ceiling height. 

All the BLE beacons were configured with the iBeacon format with a transmission interval of 200 ms, but the transmission power of the beacons has different values for each scenario. Besides, the scan window on the laptop (used to build each fingerprint vector) was set to 2 s. Therefore, during 2 s, the maximum number of advertisements that can be received from a beacon is 10. This simple data was used to check the program code and that each fingerprint is well-formed and calculated. The RSSI in dBm from each beacon is the average calculated from the advertisements received during the fingerprint interval.

In the calibration phase, a Dell laptop was used as the reference device to generate the calibration fingerprints. For performance evaluation purposes, several measurement campaigns were carried out on four different days, where testing fingerprints were generated using the same Dell laptop and a Raspberry Pi 4. Notice that the session tests were performed at the same time for both devices (the laptop and the Raspberry Pi) to have equal environment conditions, mainly to consider the RF spectrum from surrounding Wi-Fi networks and people activity. Our aims are (1) to evaluate the system performance with different BLE scanning hardware, and (2) to evaluate how the performance may change over time. The results, obtained for each scenario, are described below.

### 3.1. Scenario Terraced House

**Figure 4:**
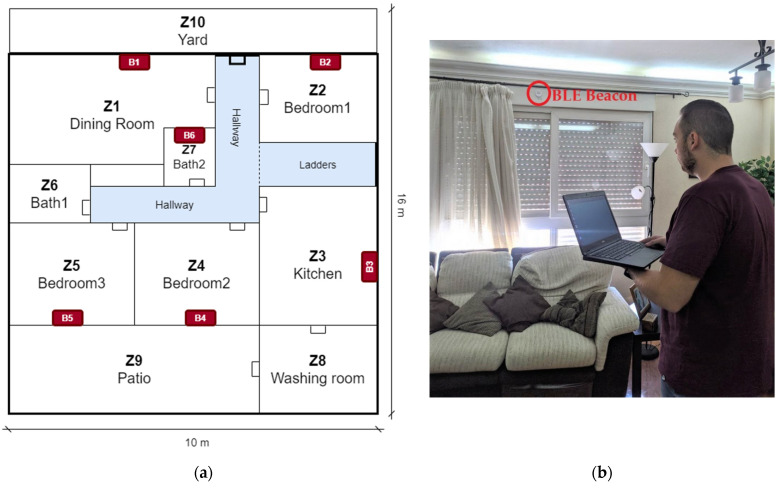
(**a**) Layout from the terraced house; (**b**) Zone 1 (dining room) with beacon B1 and user performing a test session.

The first scenario is a common example of a family home where a room-level localization system can be applied, improving the smart home concept. As can be seen in Figure 4, the terraced house has a surface of 160 m^2^ and it is divided into 10 zones according to its rooms, labeled from Z1 to Z10. Following the methodology explained before, 6 BLE beacons were deployed trying to cover the largest possible area of coverage (labeled from B1 to B6 on the map). iBKS beacons used for this experiment provide eight different transmission power levels, from −30 to +4 dBm. After a preliminary coverage analysis, all the BLE beacons were configured with a transmission power of −4 dBm. This intermediate power allows covering all the areas of the house without too much spectral overlap between beacons. It took about 10 min to place the BLE beacons according to the map. 

#### 3.1.1. Calibration Phase at Terraced House Scenario

The calibration process was carried out with the laptop. During that process, the user tried to reproduce a normal walk through each room; at each zone fingerprints were captured for 2 min, labeling them with the ID room. It took less than half an hour to calibrate all 10 zones and to generate the calibration fingerprint files.

#### 3.1.2. Testing Phase for Performance Evaluation at the Terrace House Scenario

For performance evaluation, several measurements were performed with the laptop and Raspberry at the same time on four different days of the week. During the session test of a day, the testing fingerprints per zone were registered for a min. As the fingerprint interval is set to two seconds, 30 fingerprints per zone and device were recorded. Notice that it takes less than 15 min to perform a complete walkthrough survey of all the areas of the house. All the session tests were then concatenated into a single file, called testing fingerprints, with the name of the device (laptop or Raspberry). Consequently, there are 120 testing fingerprints per room and device. Next, the testing fingerprints were processed sequentially by the room estimation engine, always using as a reference the calibration fingerprint obtained from the laptop. Hence, the results files from the session tests are obtained, and finally, the performance module calculates the related classification metrics, as discussed below.

#### 3.1.3. Beacon Mask Selection 

One important goal is to analyze the relationship between the selection and number of BLE beacons used in the fingerprint vector, i.e., the selected features of the signal pattern, and how does it affect the classification metrics. Analyzing the map layout and the deployed beacons, several combinations of BLE beacons are defined by means of the following masks:

- Mask 1: B1-B2-B3-B4-B5-B6;
- Mask 2: B1-B2-B3-B4-B5;
- Mask 3: B1-B2-B3-B5;
- Mask 4: B1-B2-B4;
- Mask 5: B3-B5-B6;
- Mask 6: B4-B5-B6;
- Mask 7: B4-B6.

This configuration of masks is aimed to cover different situations. For example, mask 1 considers a fingerprint vector with six elements, i.e., all the beacons in the house scenario. On the other hand, mask 3 considers a fingerprint vector of four elements, which are composed of the signal contributions of beacons B1, B2, B3, and B5. Beacons B4 and B6 are not considered in the fingerprint signal features evaluated under this mask. Moreover, three combinations of three beacons were defined (masks 4, 5, and 6); the purpose is to evaluate whether high accuracy performance is achieved with just three beacons (minimum required for triangulation). Finally, mask 7 is defined with only two beacons, intended to analyze a worst-case performance. 

#### 3.1.4. Accuracy and Macro-F1 Metrics in the House Scenario

**Figure 5:**
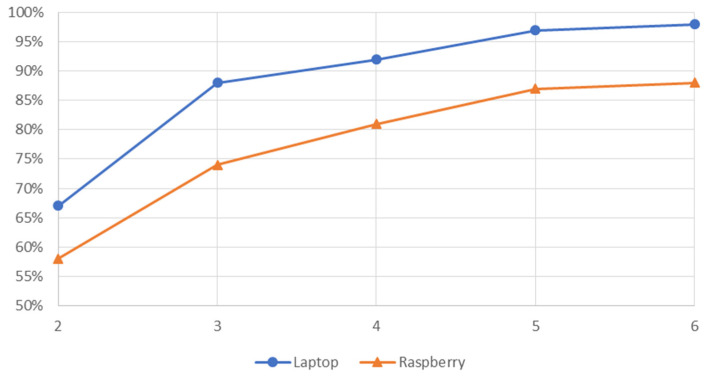
Accuracy versus number of beacons in the house scenario.

Once the masks were selected, the accuracy and macro-f1 were evaluated for each one of these combinations of beacons. For this purpose, the room estimation was carried out on the same test files for Raspberry and laptop. The results are shown in Table 2. This table shows that, for both devices, the system accuracy and macro-F1 are linearly related and are almost identical even when the number of beacons is reduced. This represents a good compromise between precision and recall metrics at room (class) level, providing a good performance of the system. Figure 5 depicts that trend for both devices.

Table 2 also shows that the overall accuracy decreases as the number of beacons in the system decreases, as expected. Firstly, it can be confirmed that the difference between a mask with five and six beacons is negligible since with both devices the accuracy is reduced by 1%. This means that the maximum recommended number of beacons in this scenario is five since from this number onwards identical results are obtained when estimating a room. 

On the other hand, using the selected combination of four BLE beacons, there is an accuracy reduction of 5–6% in both devices compared to the use of five beacons. Similarly, there is an average drop of 5–6% if a combination of three BLE beacons are used, a quite coherent value considering that an average accuracy of 87% is obtained in the case of the laptop, and 74% in the case of the Raspberry Pi just using half of the available beacons. 

It can also be seen that the accuracy drops abruptly when the two BLE beacon mask is used, obtaining a reduction of around 20% of the overall accuracy of the system. This means that the minimum number of beacons to be installed in this scenario should be three, since installing fewer beacons would have a drastic impact on the system error.

Finally, Table 2 reveals that the overall accuracy and macro-f1 obtained with the Raspberry are lower than the laptop. As expected, this is due to the fact that the calibration was performed only with the laptop, yielding worse results when using different testing hardware, such as the Raspberry Pi in this case. However, still, the trend is to achieve a high level of accuracy in spite of using different scanning hardware.

#### 3.1.5. Analyzing Room Performance Metrics

In this subsection, the main performance metrics are studied for a selection of four beacons (Mask 3: B1-B2-B3-B5), and the best combination of three beacons (Mask 4: B1-B2-B4). In order to clarify the behavior of the kNN classifier with these masks, their confusion matrices were obtained and represented in Table 3 and Table 4. 

Observing these confusion matrices, it can be seen that although only three or four beacons were considered, the kNN classifier performs reasonably well: both matrices have the higher values in the diagonal, while, the rest of the elements are zero or a very low value. 

Regarding the metrics obtained from that confusion matrices, Table 5 and Table 6 show the precision, recall, and F1-score metrics per zone for the three and four beacon masks for the laptop. Comparing both tables, it can be seen that all the metrics present values very close for all the zones, except the recall value of Z9, which is considerably affected, falling from 0.925 to 0.608. This means that, of all the existing Z9 fingerprints, only 60.8% have been correctly estimated. This also means that the F1 score decreases sharply for this zone. This was predictable since in zones Z8 and Z9, mask 4 (with only three beacons) are only covered by B4. As there is only one beacon, the system has insufficient information to differentiate properly between the two zones. Accordingly, the same trend can be appreciated with the Raspberry (Table 7 and Table 8 represent the same metrics for both masks). The confusion matrices (Table 3 and Table 4) can help to clarify that assumption: it easy to identify that most estimation errors in Z9 happen with Z8.

In addition, in order to properly compare the performance with these masks, it is convenient to include the performance measures of the system with all the beacons, i.e., mask 1 (see Table 9 and Table 10). Comparing Table 5, Table 6, Table 7 and Table 8 with the results obtained with mask 1 (Table 9 and Table 10), it can be seen that they do not differ much, but logically the values of mask 1 are better. As expected, the results are again slightly worse when using the Raspberry. See Table 11, which compares the accuracy and macro metrics of both devices.

### 3.2. Scenario Basement Floor from University Building

**Figure 6:**
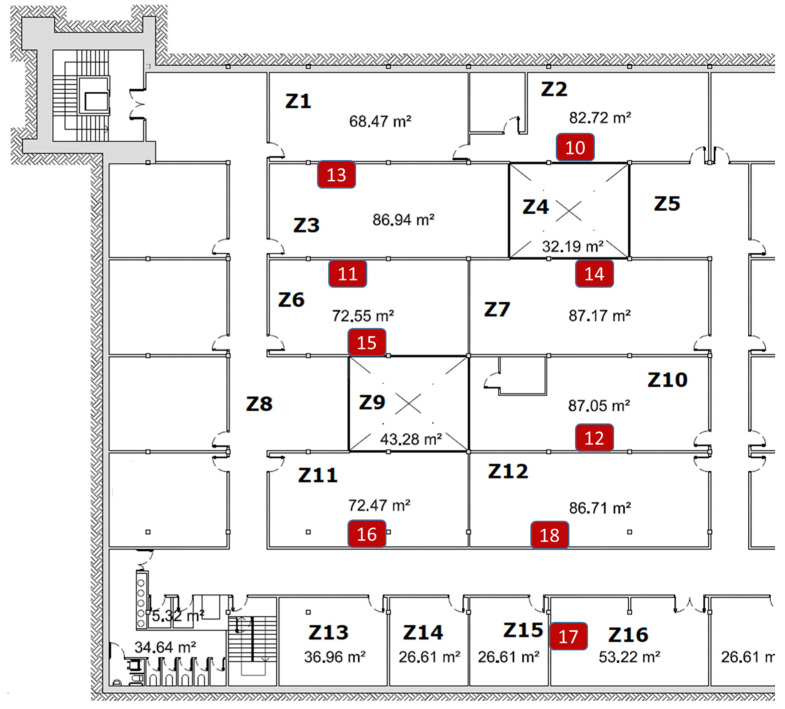
University layout. Zone labels and deployed BLE beacons.

In order to cover different scenarios, another environment is proposed. This second scenario is an area of 970 m^2^ in the basement floor at the telecommunications school building from the Technical University of Cartagena. The selected area is formed by 16 zones of different sizes, mainly labs and office rooms. Figure 6 depicts the map of that scenario where the zones have been labeled from Z1 to Z16. Notice that the term zone is used equivalent to room at the house use case. This scenario is interesting for a use case of occupancy detection of university labs, or a use case of climate and light control for energy saving purposes. 

As in the previous scenario, iBKS beacons were used, which can be configured with eight different transmission power levels, from −30 to +4 dBm. After a preliminary coverage analysis in this scenario, the transmission power for all the BLE beacons was set to +4 dBm. Unlike the previous experiment, since the areas to be covered are larger, the maximum transmission power can be used because spectral overlap does not cause problems.

#### 3.2.1. Calibration and Testing Phases for Performance Evaluation at University Scenario

As in the previous scenario, the same laptop and equal procedure for the calibration phase was used. The calibration lasted around 40 min. Then, during four days, the test measurements were performed during 1 min per zone with both laptop and Raspberry. Each testing measurement campaign lasted on average less than 25 min for the 16 zones. Finally, the testing measurements were concatenated resulting in a CSV testing file with 120 fingerprint vectors per zone and device.

#### 3.2.2. Beacon Mask Selection

In this scenario, the system performance was also analyzed when different combinations of beacons (masks) were used. Considering the map and the deployed beacons, different combinations of BLE beacons were defined using the following masks:

- Mask1: B10-B11-B12-B13-B14-B15-B16-B17-B18-B19;
- Mask2: B10-B11-B12-B13-B14-B15-B16 -B18-B19;
- Mask3: B10- B12-B13-B14-B15-B16-B18-B19;
- Mask4: B10- B12-B13-B14-B15-B16-B19;
- Mask5: B12-B13-B14-B15-B16-B19;
- Mask6: B10-B11-B16-B17-B18;
- Mask7: B10-B11-B16-B17-B19;
- Mask8: B11-B14-B16-B17-B18;
- Mask9: B11-B14-B16-B18;
- Mask10: B10-B12-B15-B16;
- Mask11: B10-B12-B15;
- Mask12: B11-B14-B17;
- Mask13: B10-B13-B17;
- Mask14: B11-B17.

This configuration of masks was also aimed to cover different situations: from all the beacons (mask1) to only two beacons (mask14) to analyze a worst-case performance.

#### 3.2.3. Accuracy and Macro-F1 Metrics in the University Scenario

With this set of masks, the overall accuracy, and macro-F1 of the system was evaluated using the estimated and true position of all the samples stored in the test files previously generated from the Raspberry and the laptop. The results are shown in Table 12.

**Figure 7:**
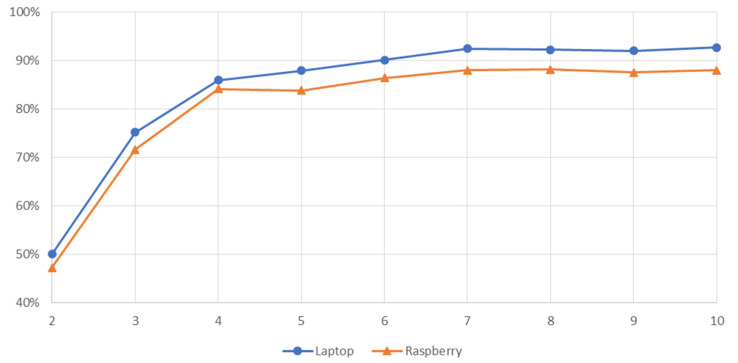
Accuracy versus number of beacons in the university scenario.

As expected, generally speaking, the more beacons used, the better the metrics obtained. However, this trend is not linear, there is no difference between masks 1, 2, and 3, and the accuracy in masks 4 and 5 hardly decreases. Complementary, Figure 7 depicts that trend for both devices. This suggests that it is possible to find a placement of beacons that minimizes the number of them while achieving high accuracy.

#### 3.2.4. Analyzing Zone Performance Metrics in the University Scenario

In the following paragraphs, the main performance metrics are studied for a selection of four beacons (Mask 9: B11-B14-B16-B18) and three beacons (Mask 12: B11-B14-B17). The confusion matrices are not included in this scenario since their relationship with the main metrics (precision, recall and F1-score) was already explained in the previous scenario. Table 13 and Table 14 show the values of these metrics for both masks with the laptop, while Table 15 and Table 16 contain the values for the Raspberry.

Table 13 and Table 14 show that the performance metrics from zones 13, 14, 15, and 16 are considerably reduced using mask 12 (B11-B14-B17). This is because these four zones (which are at the edge of the map) have only one beacon in coverage (B17) with this mask, which causes that there is no distinction among the fingerprints when the device moves to the left or right of this beacon.

Comparing these results with those obtained with mask 1 (Table 17 shows the metrics with all beacons), it can be seen that now, in some zones the difference is higher than in the other scenario, but in other zones it is not significant. 

Again, as expected, the results are slightly worse when using the Raspberry. See Table 15, Table 16, Table 18 and Table 19 (which compares the accuracy and macro metrics of both devices).

## 4. Discussion

Analyzing the data from Table 2 and Table 12, and Figure 5 and Figure 7, it can be confirmed that the minimum number of beacons required in any scenario for minimal system performance is three beacons, thereby satisfying the triangulation condition.

Moreover, it is important that these three beacons cover the entire area of the stage, adjusting the transmission power of the beacons in order to accomplish that goal. In case this condition is not fulfilled the results can be worse. For example, it is possible that in the areas located at the extremes of the scenario, the results are worse than for the central areas (where there is always three beacon coverage), as has happened in Z9 in the housing scenario and in zones 13–15 of the university basement scenario.

On the other hand, looking at the accuracy results for the family home scenario, it can be concluded that the minimum number of beacons coincides with the optimal number of beacons, since it has a high accuracy for this configuration. However, for the university basement scenario, it can be concluded that the optimal number of beacons for the system to meet minimum localization requirements is four beacons. This difference is due to the large surface area of scenario 2 (compared to scenario 1), being impossible to cover the entire area (970 m^2^) with only three beacons.

With regard to diversity across different hardware, it was verified with the use of a Raspberry Pi that the localization system responds correctly even when using different hardware from the hardware used for the calibration, obtaining a small reduction in the overall accuracy but maintaining linearity with its F1-score metrics.

Comparing the accuracy of the system between the two scenarios, it can be seen that the home case obtains a higher accuracy than the university basement. This is due to the fact that for both scenarios the tests were performed on different days in order to check the robustness of the system over time. In the home case, all the tests showed a constant value of accuracy, so it can be argued that the system performs satisfactorily against the variability of the spectrum over time for this scenario.

On the other hand, in the case of the basement, it was observed during the test measurements that the radio spectrum was much more variable than in the case of the home. This is due to the presence of several Wi-Fi access points broadcasting in the 2.4 GHz band, which also adapt their transmission power automatically, resulting in a continuous variability of the spectrum. In the case of the family home, there was only one residential router emitting in the 2.4 GHz band, so interference was very low.

Aiming to optimize the performance of the kNN-based room estimation classifier, there must be enough calibration fingerprints. From our experiments, a minimum calibration time of 2 min per zone or room was found, thus producing about 60 calibration fingerprints for each zone and fingerprints recorded by the BLE scanner with a time interval of two seconds. A minimum calibration time of at least 2 min is a trade-off between speeding the calibration process and achieving a reasonable high accuracy performance. Nevertheless, increasing the calibration time per room does not imply always an accuracy increase. We have to consider that the system approach has its limits, and it depends on the use case scenario, the number of BLE beacons, and its optimal placement. Finally, as a lesson learned to validate the performance properly, it is important to reproduce the usual mobility pattern and behavior of a person during a test session.

## 5. Conclusions

In this research, the foundations of a simple and low-cost room-level positioning system based on Bluetooth Low Energy were established. The successful room estimation of a person carrying a BLE enabled device related to the number of deployed BLE beacons was analyzed. It was empirically demonstrated that with the proper placement of a minimum number of beacons a high success rate can be achieved. 

As future work, we consider using smartphones as BLE scanners, and performance of new measurements and evaluations with rooms with medium and high occupation. Furthermore, it would be of interest to lower the cost and manpower of a BLE beacon RF planning tool to simulate the scenario and decide the number and where to place the beacons.

## Figures and Tables

**Table 1:** CSV calibration fingerprint table example scenario 1.

#	RSSI B1	RSSI B2	RSSI B3	RSSI B4	RSSI B5	RSSI B6	Dev. ID	Room ID
**1**	−90	−92	−80	−63	−70	−77	1	4
**2**	−87	−100	−74	−62	−69	−80	1	4
**3**	−72	−85	−66	−60	−56	−75	1	3
**4**	−80	−80	−65	−62	−57	−87	1	3

**Table 2:** Accuracy and macro-F1 related to the selected beacons at the fingerprints.

	Laptop		Raspberry	
	Accuracy	Macro-F1	Accuracy	Macro-F1
Mask 1: B1-B2-B3-B4-B5-B6	0.976	0.976	0.877	0.875
Mask 2: B1-B2-B3-B4-B5	0.969	0.969	0.869	0.869
Mask 3: B1-B2-B3-B5	0.918	0.918	0.809	0.807
Mask 4: B1-B2-B4	0.856	0.855	0.758	0.757
Mask 5: B3-B5-B6	0.855	0.853	0.715	0.719
Mask 6: B4-B5-B6	0.883	0.882	0.739	0.736
Mask 7: B4-B6	0.673	0.668	0.582	0.583

**Table 3:** Testing data from laptop. Confusion matrix with fingerprint vectors for mask 3 (four beacons).

	Z1	Z2	Z3	Z4	Z5	Z6	Z7	Z8	Z9	Z10
**Z1**	113	0	0	0	0	0	5	0	0	2
**Z2**	0	112	0	0	0	0	2	0	0	6
**Z3**	0	0	110	8	0	0	2	0	0	0
**Z4**	0	0	9	110	1	0	0	0	0	0
**Z5**	0	0	0	7	113	0	0	0	0	0
**Z6**	1	0	0	0	8	111	0	0	0	0
**Z7**	4	0	0	0	0	0	116	0	0	0
**Z8**	0	0	0	1	0	0	0	93	26	0
**Z9**	0	0	0	0	1	0	0	8	111	0
**Z10**	4	3	0	0	0	0	0	0	0	113

**Table 4:** Testing data from laptop. Confusion matrix with fingerprint vectors for mask 4 (three beacons).

	Z1	Z2	Z3	Z4	Z5	Z6	Z7	Z8	Z9	Z10
**Z1**	112	0	0	0	0	2	1	0	0	5
**Z2**	0	113	0	0	0	0	5	0	0	2
**Z3**	0	0	103	5	2	0	2	2	6	0
**Z4**	0	0	24	96	0	0	0	0	0	0
**Z5**	0	0	8	2	105	2	0	1	2	0
**Z6**	0	0	0	0	1	118	1	0	0	0
**Z7**	5	0	0	0	0	5	110	0	0	0
**Z8**	0	0	2	0	0	0	0	96	22	0
**Z9**	0	0	2	10	4	0	0	24	80	0
**Z10**	3	1	0	0	0	0	0	0	0	116

**Table 5:** Testing data from laptop. Classification metrics per room with fingerprint vectors with mask 3 (four beacons).

	Z1	Z2	Z3	Z4	Z5	Z6	Z7	Z8	Z9	Z10
Precision	0.926	0.974	0.924	0.873	0.919	1.000	0.928	0.921	0.810	0.934
Recall	0.942	0.933	0.917	0.917	0.942	0.925	0.967	0.775	0.925	0.942
F1-score	0.934	0.953	0.920	0.894	0.930	0.961	0.947	0.842	0.864	0.938

**Table 6:** Testing data from laptop. Classification metrics per room with fingerprint vectors with mask 4 (three beacons).

	Z1	Z2	Z3	Z4	Z5	Z6	Z7	Z8	Z9	Z10
Precision	0.941	0.974	0.707	0.836	0.927	0.885	0.930	0.736	0.709	0.942
Recall	0.925	0.95	0.883	0.767	0.85	0.967	0.892	0.767	0.608	0.95
F1-score	0.933	0.962	0.785	0.800	0.887	0.924	0.911	0.751	0.655	0.946

**Table 7:** Testing data from Raspberry. Classification metrics per room with fingerprint vectors with mask 3 (four beacons).

	Z1	Z2	Z3	Z4	Z5	Z6	Z7	Z8	Z9	Z10
Precision	0.971	0.967	0.681	0.704	0.690	0.867	0.835	0.771	0.727	0.944
Recall	0.85	0.967	0.817	0.575	0.892	0.708	0.883	0.617	0.8	0.983
F1-score	0.906	0.967	0.743	0.633	0.778	0.779	0.858	0.685	0.762	0.963

**Table 8:** Testing data from Raspberry. Classification metrics per room with fingerprint vectors with mask 4 (three beacons).

	Z1	Z2	Z3	Z4	Z5	Z6	Z7	Z8	Z9	Z10
Precision	0.969	0.966	0.541	0.730	0.741	0.802	0.853	0.604	0.604	0.841
Recall	0.783	0.95	0.6	0.542	0.833	0.842	0.775	0.725	0.558	0.967
F1-score	0.866	0.958	0.569	0.622	0.784	0.822	0.812	0.659	0.580	0.900

**Table 9:** Testing data from laptop. Classification metrics per room with fingerprint vectors with all the beacons.

	Z1	Z2	Z3	Z4	Z5	Z6	Z7	Z8	Z9	Z10
Precision	0.975	1	0.967	0.975	0.983	1	0.975	1	0.8950	1
Recall	0.975	1	0.992	0.975	0.983	0.992	0.992	0.875	0.992	0.983
F1-score	0.975	1	0.979	0.975	0.983	0.996	0.983	0.933	0.941	0.991

**Table 10:** Testing data from Raspberry. Classification metrics per room with fingerprint vectors with all the beacons.

	Z1	Z2	Z3	Z4	Z5	Z6	Z7	Z8	Z9	Z10
Precision	1	0.992	0.684	0.821	0.812	0.932	0.974	0.85	0.833	0.93
Recall	0.883	0.983	0.883	0.575	0.9	0.917	0.95	0.8	0.875	1
F1-score	0.938	0.987	0.771	0.676	0.854	0.924	0.962	0.824	0.853	0.964

**Table 11:** House scenario performance metrics with fingerprint vectors with all the beacons.

Mask1: All the Beacons	Laptop	Raspberry pi
Accuracy	0.976	0.877
Macro-Precision	0.977	0.883
Macro-Recall	0.976	0.877
Macro-F1	0.976	0.875

**Table 12:** Accuracy and macro-F1 related to the selected beacons at the fingerprints.

	Laptop		Raspberry	
	Accur.	M-F1	Accur.	M-F1
Mask 1: B10-B11-B12-B13-B14-B15-B16-B17-B18-B19	0.927	0.926	0.880	0.879
Mask 2: B10-B11-B12-B13-B14-B15-B16 -B18-B19	0.920	0.919	0.875	0.874
Mask 3: B10- B12-B13-B14-B15-B16-B18-B19	0.922	0.921	0.881	0.880
Mask 4: B10- B12-B13-B14-B15-B16-B19	0.924	0.923	0.880	0.879
Mask 5: B12-B13-B14-B15-B16-B19	0.901	0.900	0.864	0.863
Mask 6: B10-B11-B16-B17-B18	0.865	0.864	0.835	0.834
Mask 7: B10-B11-B16-B17-B19	0.879	0.878	0.838	0.837
Mask 8: B11-B14-B16-B17-B18	0.866	0.865	0.826	0.824
Mask 9: B11-B14-B16-B18	0.815	0.812	0.796	0.792
Mask 10: B10-B12-B15-B16	0.859	0.859	0.841	0.841
Mask 11: B10-B12-B15	0.752	0.744	0.716	0.726
Mask 12: B11-B14-B17	0.696	0.691	0.672	0.664
Mask 13: B10-B13-B17	0.663	0.648	0.651	0.641
Mask 14: B11-B17	0.501	0.487	0.473	0.459

**Table 13:** Testing data from laptop. Classification metrics per zone with fingerprint vectors with mask 9 (four beacons).

	Z1	Z2	Z3	Z4	Z5	Z6	Z7	Z8	Z9	Z10	Z11	Z12	Z13	Z14	Z15	Z16
Precision	1	0.83	0.642	0.702	0.798	0.659	0.796	0.944	0.59	0.904	0.958	0.846	0.962	0.863	0.913	0.991
Recall	0.717	0.65	0.583	0.825	0.658	0.95	0.942	0.425	0.925	0.858	0.958	0.917	0.833	0.942	0.958	0.9
F1-score	0.835	0.729	0.611	0.759	0.721	0.778	0.863	0.586	0.72	0.88	0.958	0.88	0.893	0.901	0.935	0.943

**Table 14:** Testing data from laptop. Classification metrics per zone with fingerprint vectors with mask 12 (three beacons).

	Z1	Z2	Z3	Z4	Z5	Z6	Z7	Z8	Z9	Z10	Z11	Z12	Z13	Z14	Z15	Z16
Precision	1	0.639	0.742	0.705	0.873	0.68	0.698	0.652	0.708	0.719	0.92	0.837	0.508	0.361	0.567	0.88
Recall	0.758	0.65	0.742	0.817	0.458	0.958	0.925	0.625	0.95	0.833	0.667	0.6	0.75	0.292	0.567	0.55
F1-score	0.862	0.644	0.742	0.757	0.601	0.795	0.796	0.638	0.811	0.772	0.773	0.699	0.606	0.323	0.567	0.677

**Table 15:** Testing data from Raspberry. Classification metrics per zone with fingerprint vectors with mask 9 (four beacons).

	Z1	Z2	Z3	Z4	Z5	Z6	Z7	Z8	Z9	Z10	Z11	Z12	Z13	Z14	Z15	Z16
Precision	0.927	0.775	0.816	0.727	0.71	0.728	0.743	0.929	0.576	0.852	0.929	0.853	0.922	0.78	0.812	0.935
Recall	0.85	0.658	0.667	0.8	0.55	0.892	0.942	0.433	0.95	0.908	0.875	0.825	0.792	0.858	0.9	0.833
F1-score	0.887	0.712	0.734	0.762	0.62	0.802	0.831	0.591	0.717	0.879	0.901	0.839	0.852	0.817	0.854	0.881

**Table 16:** Testing data from Raspberry. Classification metrics per zone with fingerprint vectors with mask 12 (three beacons).

	Z1	Z2	Z3	Z4	Z5	Z6	Z7	Z8	Z9	Z10	Z11	Z12	Z13	Z14	Z15	Z16
Precision	1	0.639	0.742	0.705	0.873	0.68	0.698	0.652	0.708	0.719	0.92	0.837	0.508	0.361	0.567	0.88
Recall	0.758	0.65	0.742	0.817	0.458	0.958	0.925	0.625	0.95	0.833	0.667	0.6	0.75	0.292	0.567	0.55
F1-score	0.862	0.644	0.742	0.757	0.601	0.795	0.796	0.638	0.811	0.772	0.773	0.699	0.606	0.323	0.567	0.677

**Table 17:** Testing data from laptop. Classification metrics per zone with fingerprint vectors with all the beacons.

	Z1	Z2	Z3	Z4	Z5	Z6	Z7	Z8	Z9	Z10	Z11	Z12	Z13	Z14	Z15	Z16
Precision	1	0.978	0.854	0.745	0.95	0.908	0.93	0.99	0.816	0.99	1	0.944	1	0.895	0.984	1
Recall	0.842	0.733	0.975	1	0.8	0.983	0.992	0.808	1	0.842	0.992	0.992	0.883	0.992	1	0.992
F1-score	0.914	0.838	0.91	0.854	0.869	0.944	0.96	0.89	0.899	0.91	0.996	0.967	0.938	0.941	0.992	0.996

**Table 18:** Testing data from Raspberry Pi. Classification metrics per zone with fingerprint vectors with all the beacons.

	Z1	Z2	Z3	Z4	Z5	Z6	Z7	Z8	Z9	Z10	Z11	Z12	Z13	Z14	Z15	Z16
Precision	0.945	0.927	0.867	0.712	0.84	0.835	0.889	0.924	0.786	0.916	0.958	0.949	0.972	0.86	0.869	0.947
Recall	0.858	0.742	0.925	0.967	0.658	0.925	0.933	0.708	0.95	0.908	0.942	0.925	0.875	0.925	0.942	0.892
F1-score	0.899	0.824	0.895	0.82	0.738	0.878	0.91	0.802	0.86	0.912	0.95	0.937	0.921	0.891	0.904	0.919

**Table 19:** University scenario performance metrics with fingerprint vectors with all the beacons.

Mask1: All the Beacons	Laptop	Raspberry Pi
Accuracy	0.927	0.880
Macro-Precision	0.937	0.887
Macro-Recall	0.927	0.880
Macro-F1	0.926	0.879

## Data Availability

Data is available at http://doi.org/10.5281/zenodo.4783076.
